# The risk and survival outcome of subsequent primary colorectal cancer after the first primary colorectal cancer: cases from 1973 to 2012

**DOI:** 10.1186/s12885-017-3765-8

**Published:** 2017-11-22

**Authors:** Jiao Yang, Xianglin L. Du, Shuting Li, Yinying Wu, Meng Lv, Danfeng Dong, Lingxiao Zhang, Zheling Chen, Biyuan Wang, Fan Wang, Yanwei Shen, Enxiao Li, Min Yi, Jin Yang

**Affiliations:** 1 Departments of Medical Oncology, The First Affiliated Hospital of Xi’an Jiaotong University, 277 Yanta West Road, Xi’an, 710061 Shaanxi Province People’s Republic of China; 2 Division of Epidemiology & Disease Control, School of Public Health, The University of Texas Health Science Center at Houston, Houston, TX USA; 3 Breast Surgical Oncology, The University of Texas MD Anderson Cancer Center, Houston, TX USA

**Keywords:** Subsequent primary colorectal cancer, Tumor location, Age

## Abstract

**Background:**

Among colorectal cancer (CRC) survivors, how the prior tumor location affects the risk of subsequent primary colorectal cancer (SPCRC) and the outcome of those suffering from SPCRC remain unknown.

**Methods:**

CRC cases diagnosed from 1973 to 2012 were screened for SPCRC development using the Surveillance, Epidemiology, and End Results database. The relative risk of SPCRC was estimated using the standardized incidence ratio. Survivals were analyzed using the Kaplan–Meier and Cox regression model.

**Results:**

The overall risk of SPCRC increased by 27% in CRC survivors compared to that of the general population. The risk increased in patients with both prior right colon cancer (RCC) and left colon cancer (LCC), and was concentrated in the first 5 years after the prior diagnosis, and among young patients. Among the 6701 SPCRC patients identified, patients with prior RCC were more likely to be elderly, female, and with more low or undifferentiated disease than those with prior LCC or rectal cancer (ReC). The overall survivals differed by both prior tumor location (*P* < 0.0001) and age (*P* < 0.0001), and the difference by tumor location remained significant when adjusted or stratified by any other potential prognostic factor except age. The cancer specific survivals differed by age (*P* < 0.0001) rather than by prior tumor location (*P* = 0.455).

**Conclusions:**

The overall risk of SPCRC increased among patients with both prior RCC and LCC, but not among those with ReC. The different survival outcomes in CRC survivors suffering from SPCRC were largely explained by the patient age but not by the prior tumor location.

**Electronic supplementary material:**

The online version of this article (10.1186/s12885-017-3765-8) contains supplementary material, which is available to authorized users.

## Background

Colorectal cancer (CRC) is the third most common cancer worldwide and the second most common cause of cancer-related deaths in Western countries. The survival of CRC patients has improved gradually with the widespread use of advanced diagnostic and therapeutic methods, including fecal occult bleeding tests, colonoscopy screening, targeted treatments, and multidisciplinary team therapy approaches. The increased survival rate has resulted in increased risks of developing subsequent primary malignancies, among which the subsequent primary colorectal cancer (SPCRC) was the most commonly observed form of cancer [1, 2]. Synchronous colorectal adenoma and family history of CRC are indicators for SPCRC [3–5]. Furthermore, the risk of SPCRC was reported to change over time and was influenced by other factors, including latency and age [6–8].

Tumors derived from different colorectal segments have distinct clinicopathological features and genetic variations*.* The risk of SPCRC was shown to differ by tumor location prior to CRC, but the studies remain controversial. Some studies indicated a high risk in patients with prior cancer located in the proximal colon [9, 10]. Gervaz et al. [9] proposed that the proximal location of colon cancer was a risk factor for SPCRC based on a higher prevalence of SPCRC in patients with prior right colon cancer (RCC; 3.4%) than those with prior left colon cancer (LCC), and rectal cancer (ReC; 1.8%) after long-term follow-up. However, other studies suggested a high risk in those patients with prior cancer located in the distal colon or rectum [11–13]. For example, Borda et al. [12] found that SPCRC developed more frequently among patients with prior cancer located distally rather than proximally [odds ratio (OR) = 2.30]. Hence, the influence of prior tumor location on the risk of SPCRC needs to be validated by large-scale studies.

In addition, tumor location was reported to influence the survival outcomes of patients with single CRC [14, 15]. Among patients with metastatic CRC, patients with proximal colon cancer had a worse outcome in comparison to those patients with metastatic distal colon cancer or ReC [16]. Nevertheless, data is lacking concerning the survival rates of CRC survivors suffering from SPCRC. We hypothesize that the survival of CRC patients suffering from SPCRC may differ by the tumor location of the prior CRC.

In the current study, the influence of prior tumor location on SPCRC development and on the survival of SPCRC patients was determined based on the Surveillance, Epidemiology, and End Results (SEER) database. The CRC survivors were divided into three groups according to the prior tumor location, and were designated as the RCC, LCC, and ReC groups. The influence of prior tumor location, as well as latency and age, on the risk of SPCRC was assessed. Whether tumor location of the prior CRC could predict outcomes of the patients with SPCRC was further evaluated.

## Methods

### Data source and study cohort

Cancer incidence was identified from the National Cancer Institute Surveillance, Epidemiology, and End Results (SEER) Program database. The Multiple-Primary session of the SEER*Stat software 8.2.1 was used to estimate the standardized incidence of SPCRC in CRC cases based on the SEER 9 registry data, including individual data from 1973 to 2012. To ensure that recurrences and metastases are not recorded as new primary cancers, SEER registrars adhere to a series of coding rules.

For the present study, index cases included individuals diagnosed with an index colorectal adenocarcinoma confirmed pathologically between 1973 and 2005, in order to allow at least 7 years of follow-up for screening second SPCRC and studying outcomes. Cases that lacked documentation of age at diagnosis or were reported only on death certificates or autopsy reports were excluded. The inclusion criteria for screening SPCRC were as follows: (1) either in situ or malignant; (2) pathologically confirmed; (3) diagnosed subsequent to the index CRC; and (4) diagnosed on or before December 31, 2012. A minimum latency period of 6 months between the diagnoses was required to exclude synchronous cancers. In total, the final study cohort comprised 7290 patients who met the inclusion criteria for SPCRC from the pool of 202,088 index CRC cases.

### Estimation of standardized incidence ratio for SPCRC

To determine the relative risk of SPCRC among all the index CRC cases, the standardized incidence ratio (SIR) was calculated as the ratio of the observed number to the expected number of SPCRCs based on CRC incidence in the general population of the SEER ascertainment areas, with adjustment for sex, age, calendar year, and race. More information on both SEER*Stat software and the method it uses to derive the SIRs are available on the SEER website (available at http://seer.cancer.gov/resources/). SIRs were calculated for subpopulations with different anatomical sites of the prior tumor, in order to validate if it was reasonable to divide patients into RCC, LCC, and ReC groups according to prior tumor location. Then SIRs for each subgroup were calculated to assess the influence of prior tumor location, latency, and age of prior diagnosis on SPCRC development. A determination of statistical significance was based on a two-sided *p*-value < 0.05.

### Statistical analyses on clinical features

Detailed information was extracted on sex, ethnicity, stage, grade, age, and year of first diagnosis and anatomical subsites of prior tumors among the cohort. Chi-squared tests were used to assess the differences in demographic and clinical characteristics between the three location groups. The overall survival (OS) was calculated from the date of diagnosis of the prior CRC to the date of all-caused death, or last contact (if the patient was lost to follow-up), or December 31, 2012, whichever occurred first. Regarding cancer-specific survival (CSS), the event is specific to CRC-related death, instead of all-caused death. The survival rate was calculated using the Kaplan–Meier method by SPSS software, version 18.0 (SPSS, Chicago, IL, USA), and was compared using the log-rank test for significant difference by prior tumor location or patient age. Cox regression model was used to assess the independent effect of prior tumor location or age on the hazard ratio (HR) of developing SPCRC after controlling for other variables. All tests were two-tailed, and statistical significance was set at *P* < 0.05.

## Results

### Risk of SPCRC

**Fig. 1:**
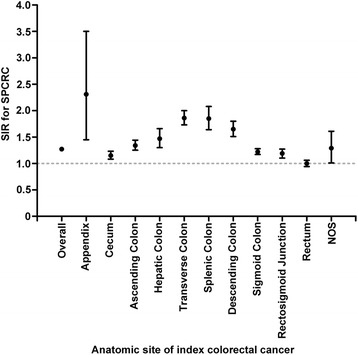
Standardized incidence ratio for SPCRC by anatomical sites of index colorectal cancer (Abbreviations: SPCRC, subsequent primary colorectal cancer; SIR, standardized incidence ratio; NOS, non-specific)

A total of 7290 cases with prior CRC were shown to develop SPCRC from 1973 to 2005. The overall risk of CRC increased by 27% (95% CI: 24%–30%) in CRC survivors compared to that of the general population (Fig. 1, Additional file 1: Table S1). The risk of SPCRC increased significantly among patients with prior colon cancer (SIRs > 1). Furthermore, when grouped by the anatomical sites of the prior tumor, the SIR increased from 1.15 at the cecum to 1.86 at the transverse colon, and then declined from 1.85 at the splenic colon to 1.19 at the rectosigmoid junction. However, the risk of SPCRC in patients with prior ReC was comparable to the risk of primary CRC in the general population (SIR = 1), and was stable throughout the extended follow-up time.

**Fig. 2:**
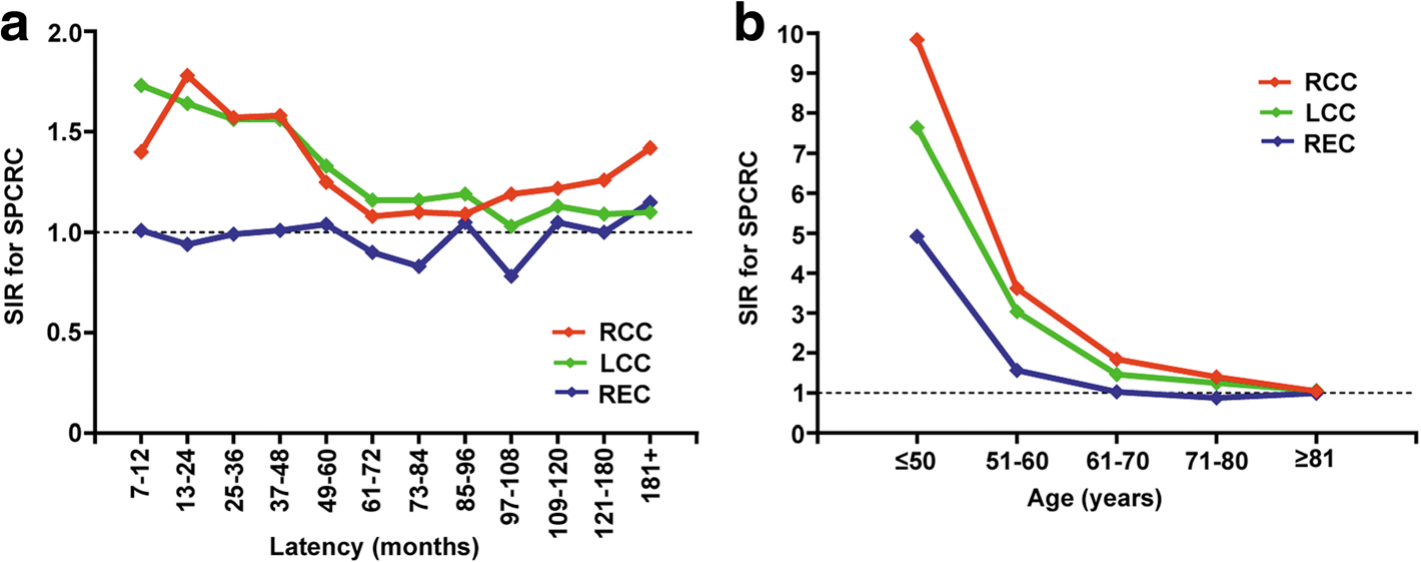
Standardized incidence ratio for SPCRC by (**a**) latency or (**b**) age among colorectal cancer survivors. (Abbreviations: SPCRC, subsequent primary colorectal cancer; SIR, standardized incidence ratio; RCC, right colon cancer; LCC, left colon cancer; ReC, rectal cancer)

Among patients with RCC or LCC, the risk of SPCRC increased in the first 60 months after prior diagnosis (Fig. 2a, Additional file 2: Table S2). Moreover, the risk remained elevated by 16%–19% at 60–96 months among the LCC patients, while the risk was still more than 20% higher after 108 months’ follow-up among the RCC patients, compared to the risk of primary CRC in the general population. The risk of SPCRC decreased significantly with increasing age (Fig. 2b, Additional file 3: Table S3). The tendency was most obvious in the RCC survivors, obvious in the LCC survivors, and then least obvious in the ReC survivors. The risks were similar to that of the general population in RCC and LCC patients at ages older than 80 years, and in the ReC patients at ages older than 60 years.

### Patient and tumor characteristics

Detail information could be retrieved for 6701 patients from the 7290 cases suffering from SPCRC. Comparison was made on clinical features of the initial CRC between the patients with single CRC and patients with SPCRC (Additional file 4: Table S4). Of the 6701 patients with SPCRC, 38.4% had prior RCC, 47.2% had prior LCC, and 14.4% had prior ReC. The median age of these patients at prior diagnosis was 69 years (range, 14–97 years). More than half of them were diagnosed with grade II tumors. The median age was higher for the RCC group (75 years) than for the LCC group (69 years) and Rec group (67 years) (Table 1). Compared to patients with prior LCC and ReC, those patients with prior RCC were more likely to be older, female, and to have more low or undifferentiated grade pathology.

**Table 1:** Characteristics of index colorectal cancer among SPCRC patients according to prior tumor location

Variable	RCC (*n* = 2572)	LCC (*n* = 3166)	ReC (*n* = 963)	P
Age at diagnosis				< 0.0001
median (range)	70 (14–97)	68 (27–96)	67 (22–96)	
≤ 50	194 (7.5)	209 (6.6)	71 (7.4)	
51–60	339 (13.2)	568 (17.9)	204 (21.2)	
61–70	776 (30.2)	1095 (34.6)	324 (33.6)	
71–80	883 (34.3)	997 (31.5)	270 (28.0)	
≥ 81	380 (14.8)	297 (9.4)	94 (9.8)	
Year of diagnosis				0.158
1973–1985	1273 (49.5)	1676 (53.0)	485 (50.4)	
1986–1995	886 (33.7)	1008 (31.8)	332 (34.5)	
1996–2005	433 (16.8)	482 (15.2)	146 (15.1)	
Race				0.007
white	2218 (86.2)	2729 (86.2)	828 (86.0)	
Black	214 (8.3)	211 (6.7)	62 (6.4)	
Others	140 (5.5)	206 (7.1)	73 (7.6)	
Gender				<0.0001
Female	1347 (52.4)	1389 (43.9)	402 (41.7)	
Male	1225 (47.6)	1777 (56.1)	561 (58.3)	
Stage				<0.0001
Localized	1008 (39.2)	1408 (44.4)	554 (57.5)	
Regional	1395 (54.2)	1544 (48.8)	338 (35.1)	
Distant	112 (4.4)	132 (4.2)	17 (1.8)	
Unknown	57 (2.2)	82 (2.6)	54 (5.6)	
Tumor grade				<0.0001
Grade I	307 (11.9)	438 (13.8)	123 (12.8)	
Grade II	1302 (50.6)	1797 (56.8)	535 (55.6)	
Grade III	537 (20.9)	357 (11.3)	116 (12.0)	
Unknown	426 (16.6)	574 (18.1)	189 (19.6)	

*Abbreviations*: *SPCRC* Subsequent primary colorectal cancer, *RCC* Right colon cancer, *LCC* Left colon cancer, *ReC* Rectal cancer

### Overall survival by location or age

**Fig. 3:**
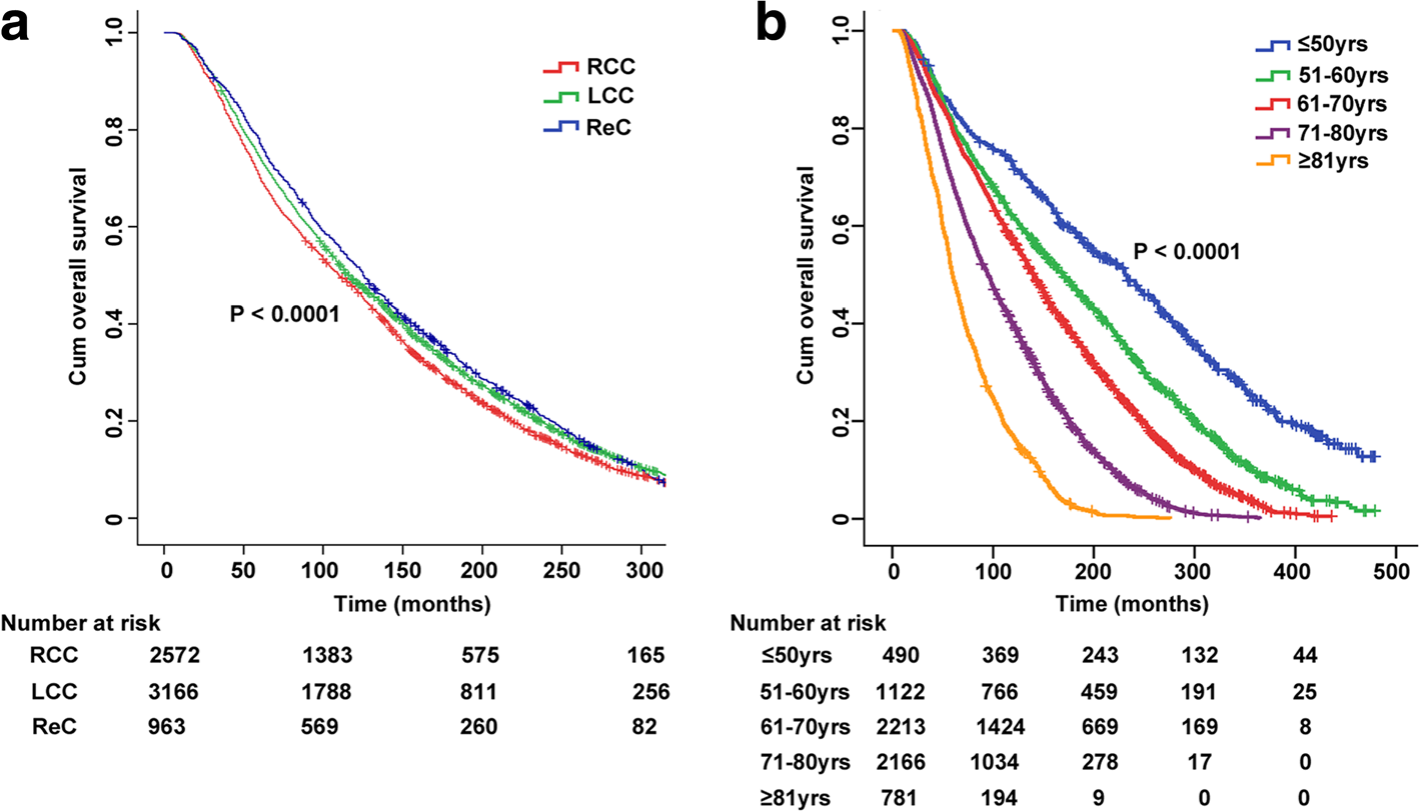
Overall survival among SPCRC patients by (**a**) prior tumor location or (**b**) age at prior diagnosis. (Abbreviations: SPCRC, subsequent primary colorectal cancer; RCC, right colon cancer; LCC, left colon cancer; ReC, rectal cancer)

The entire cohort had a five-year overall survival (OS) of 73.0% and a 10 year survival of 48.6%, with a median survival time of 116 months (95% CI: 112–120). The survival percentages differed according to tumor location of the prior CRC (*P* < 0.0001) (Fig. 3a). The five-year survival percentages were 70.1%, 74.2%, and 77.1% in the RCC, LCC, and ReC groups, respectively, with corresponding median survival times of 110, 116, and 125 months, respectively. The survival of the cohort also varied according to initial age at diagnosis. The older the patient was when diagnosed with prior CRC, the worse was the outcome (*P* < 0.0001) (Fig. 3b). The five-year OS percentages were 83.9% in the ≤50 years of age group, 81.1% in the 51–60 years of age group, 79.3% in the 61–70 years of age group, 68.5% in the 71–80 years of age group, and 49.5% in the ≥81 years of age group, with corresponding median survival times of 233, 170, 140, 94, and 60 months, respectively.

### Overall survival by both location and age

In multivariate analyses (Table 2), five factors including stage, race, age, diagnosis year, and sex were indicated as independent prognostic predictors for overall survival, excluding tumor location of the prior CRC. According to stratified analyses (Table 3, Additional file 5: Table S5), the survival difference by prior tumor location within each stratified subgroup disappeared when stratified by the factor of age, but were still significant within location subgroups when stratified by other factors in stratified analyses.

**Table 2:** Cox proportional hazards analysis of overall survival and cancer-specific survival

Variable	Overall survival				Cancer-specific survival			
	HR	95% CI		P	HR	95% CI		P
Location				0.076				0.054
RCC	Referent				Referent			
LCC	0.94	0.89	0.99	0.028	1.01	0.93	1.10	0.811
Rectum	0.99	0.91	1.07	0.741	1.15	1.02	1.29	0.022
Age at diagnosis (year)				<0.0001				<0.0001
≤ 50	Referent				Referent			
51–60	1.70	1.50	1.92	<0.0001	1.27	1.07	1.51	0.007
61–70	2.35	2.08	2.63	<0.0001	1.35	1.15	1.58	<0.0001
71–80	3.91	3.46	4.40	<0.0001	1.97	1.68	2.32	<0.0001
≥ 81	7.18	6.26	8.22	<0.0001	3.23	2.68	3.91	<0.0001
Stage				<0.0001				<0.0001
Localized	Referent				Referent			
Regional	1.16	1.11	1.23	<0.0001	1.50	1.38	1.63	<0.0001
Distant	2.17	1.90	2.47	<0.0001	3.72	3.15	4.39	<0.0001
Unknown	1.21	1.04	1.40	0.014	1.48	1.19	1.85	0.001
Grade				0.949				0.491
Grade I	Referent				Referent			
Grade II	1.01	0.93	1.09	0.853	1.07	0.95	1.22	0.258
Grade III	1.02	0.93	1.12	0.700	1.12	0.96	1.30	0.148
Unknown	0.98	0.98	1.07	0.673	1.10	0.95	1.27	0.198
Race				<0.0001				<0.0001
White	Referent				Referent			
Black	1.24	1.13	1.36	<0.0001	1.31	1.14	1.51	<0.0001
Others	0.94	0.85	1.04	0.223	0.84	1.80	0.99	0.038
Gender				<0.0001				<0.0001
Male	Referent				Referent			
Female	0.74	0.70	0.78	<0.0001	0.83	0.77	0.90	<0.0001
Year of diagnosis				<0.0001				<0.0001
1973–1985	Referent				Referent			
1986–1995	1.27	1.20	1.36	<0.0001	1.24	1.13	1.36	<0.0001
1996–2005	2.07	2.91	2.51	<0.0001	2.03	1.80	2.28	<0.0001

*Abbreviations*: *HR* Hazard ratio, *CI* confidence interval

**Table 3:** Five-year survival rates of SPCRC patients according to prior tumor location in stratified subgroups

Variable	Overall survival (%)				Cancer-specific survival (%)			
	RCC	LCC	ReC	P	RCC	LCC	ReC	P
All	70.1	74.2	77.1	<0.0001	80.9	83.7	86.7	0.455
Stage								
Localized	76.4	79.3	82.5	0.022	89.3	88.8	91.0	0.115
Regional	67.9	72.4	70.1	0.010	77.7	81.8	81.1	0.143
Distant	36.6	45.5	32.1	0.775	46.1	52.2	51.0	0.929
Unknown	77.2	67.1	77.8	0.046	80.5	83.2	86.7	0.436
Grade								
I	72.3	80.8	78.9	0.693	86.3	90.5	87.8	0.901
II	67.5	71.2	75.3	0.002	79.0	81.5	85.7	0.09
III	67.2	73.7	74.1	0.143	78.7	82.0	81.2	0.294
Unknown	79.8	78.9	83.1	0.361	84.3	86.2	86.2	0.545
Race								
White	70.4	74.6	77.6	<0.0001	81.1	75.5	86.0	0.191
Black	61.7	69.2	69.4	0.687	84.1	76.9	84.4	0.150
Others	75.7	73.9	78.1	0.700	87.2	79.2	87.0	0.575
Gender								
Male	67.1	72.2	73.8	0.011	79.4	83.0	73.8	0.452
Female	72.8	76.8	81.8	0.001	82.3	84.6	88.9	0.454
Year of diagnosis								
1973–1985	77.1	80.2	84.3	0.002	85.1	87.5	90.6	0.372
1986–1995	71.2	74.0	76.5	0.768	83.0	83.6	85.1	0.514
1996–2005	47.1	53.9	54.8	0.327	63.1	69.4	75.7	0.870
Age at diagnosis								
≤ 50	83.5	81.3	90.1	0.779	87.5	83.1	91.5	0.053
51–60	76.1	80.8	87.7	0.295	82.8	86.0	92.5	0.344
61–70	77.6	80.1	80.9	0.427	85.5	87.4	89.6	0.965
71–80	67.6	68.5	71.5	0.458	80.4	81.3	83.6	0.452
≥ 81	48.2	52.2	46.8	0.225	67.4	72.0	65.8	0.470

*Abbreviations*: *SPCRC* Subsequent primary colorectal cancer, *RCC* Right colon cancer, *LCC* Left colon cancer, *ReC* Rectal cancer

### CSS by location or age

**Fig. 4:**
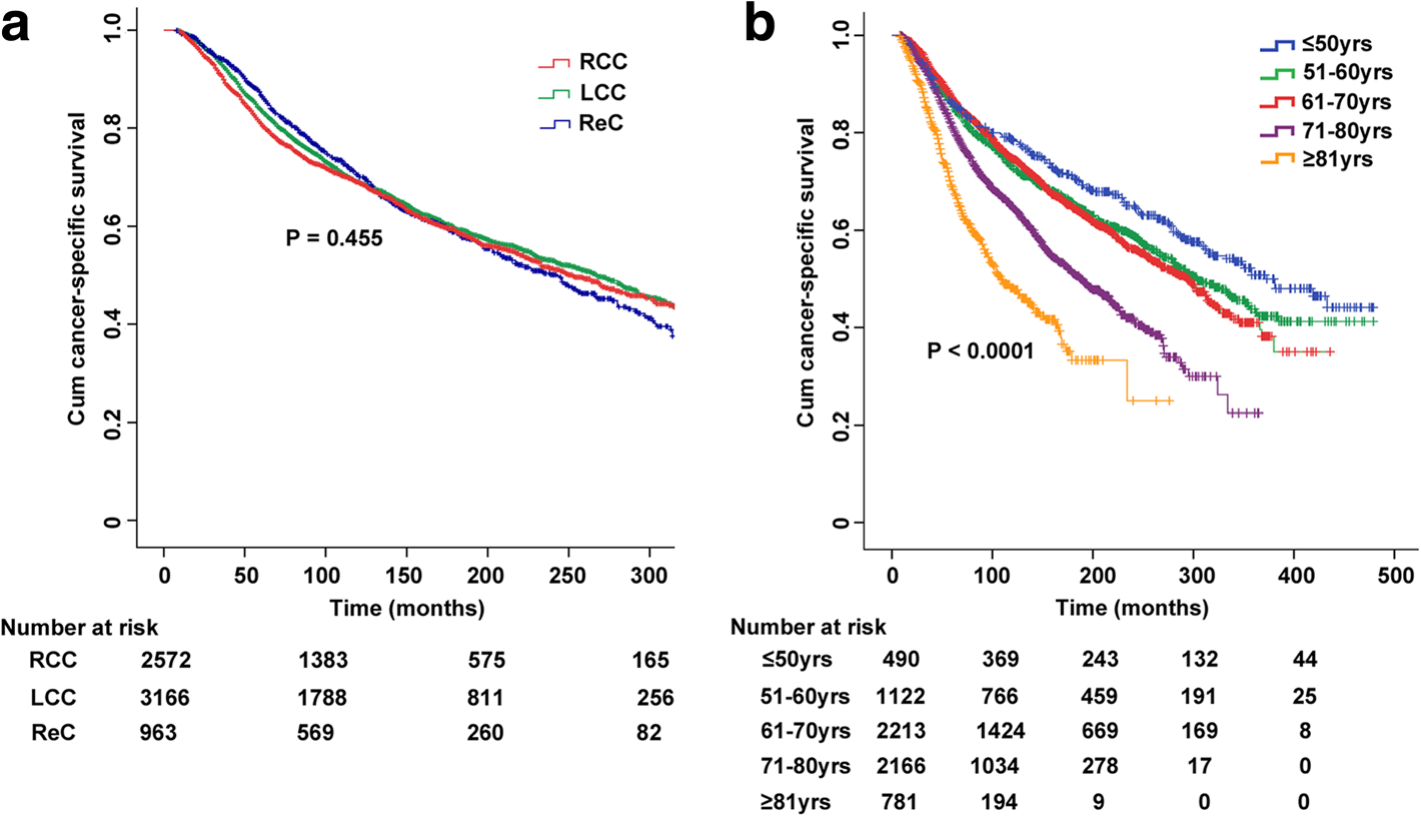
Cancer-specific survival among SPCRC patients by (**a**) prior tumor location or (**b**) age at prior diagnosis. (Abbreviations: SPCRC, subsequent primary colorectal cancer; RCC, right colon cancer; LCC, left colon cancer; ReC, rectal cancer)

Further comparisons of CSS indicated that the outcomes were similar among the three location groups (*P* = 0.455), but were significantly different among the five age groups (*P* < 0.0001) (Fig. 4, Table 2). Increasing age at diagnosis of initial CRC was associated with poorer CSS. However, no obvious difference was found between the 51–60 years of age and the 61–70 years of age groups (*P* = 0.579).

## Discussion

The current study had two major findings. First, the risk of SPCRC differed by prior tumor location as well as by age and latency. The risk of SPCRC increased both in patients with prior RCC and in patients with prior LCC, whereas, patients with ReC had similar risks in comparison to that of the general population. The increased risk of SPCRC mainly occurred within the first 5 years after prior diagnosis and among young patients. Second, the survival of patients with SPCRC did not differ significantly by initial CRC locations. Patients with SPCRC after RCC seemed to have a worse OS than those patients with SPCRC after LCC or ReC, but the survival differences by tumor locations disappeared when adjusted or stratified by age. It was age rather than tumor location of prior CRC that was recognized as an independent prognostic factor among patients with SPCRC.

Previous studies have reported that the risk of SPCRC was higher in patients with proximal or distal CRC based on the proportion of SPCRC prevalence [9, 13]. This study assessed the relative risk of SPCRC using SIR, which was more useful than the simple prevalence. The results indicated that the degree of risk increased and then declined with the cutoff of splenic flexure and with the anatomical sites of prior CRC, located from proximally to distally. This supported the division pattern of colon cancer into right and left subsides. Of significance, all patients with prior colon cancer had an increased risk of SPCRC, with no significant difference observed between the RCC and LCC groups. In agreement with the current results, a study showed that the risk of SPCRC increased by 40% among all colon cancer patients, but the risk was similar to that of the general population in ReC patients [10].

Compared to the RCC and LCC groups, ReC patients were more likely to be younger than 60 years of age and mostly had localized disease. Hence, the ReC survivors may have had a greater chance of being cured, leading to a relatively longer follow-up duration. Furthermore, much more bowel segments were left for the tumor cells to grow among the ReC survivors after radical surgery, than among those patients with prior colon cancer. However, patients with prior ReC did not have increased risks, although they seemed to have various advantages in developing SPCRC. ReC may therefore be a distinct form colon cancer.

The risk of SPCRC was obviously concentrated in the first 5 years after prior diagnoses among patients with RCC or LCC. In previous studies, the risk of SPCRC was reported to be mostly focused in the first 3 years after prior diagnoses, because more than half of the SPCRC cases developed during this time period [17, 18]. We observed that the risks of SPCRC were similar at 3–4 years. In the 5th year, these patients still had elevated risks of SPCRC, although the degree of risk increase began to decline. Five years later, the risk of SPCRC differed between patients with prior RCC and LCC. Therefore, in the process of SPCRC screening, attention should be given for at least the first 5 years after prior diagnosis among CRC survivors. The location of the prior CRC should also be taken into consideration in individual patients, using a longer surveillance strategy.

Several previous studies showed a higher risk of SPCRC among older patients than among younger patients [19–21]. The majority of the patients with SPCRC were older. This may be attributed to the high prevalence of prior CRC among the elderly patients. The longer follow-up period since prior CRC diagnosis may also have partly contributed to these results. This study showed that when patients were diagnosed with prior colon cancer at a younger age, they had higher risks of SPCRC. The results were consistent with previous studies [8, 22]. Interestingly, the risk of SPCRC was higher among patients with prior RCC than among those with prior LCC, within each age group. Therefore, the risk of SPCRC should be higher among the RCC patients than among the LCC survivors in the entire cohort. However, no significant difference was observed in the overall risk of SPCRC between these two groups. These results could be due to the different patterns of age distributions between the RCC and LCC groups. Hence, more attention should be paid to young patients with prior colon cancer at the first 5 years after prior diagnosis in clinical surveillance of SPCRC.

One of the major limitations from previous studies was the lack of information on survival outcomes of patients with SPCRC after prior CRC. In our study, a favorable OS was observed among these patients. This may be because these patients underwent a second screening by the inclusion criteria of SPCRC development. Patients with early stage disease and long follow-up times had a greater possibility of developing SPCRC. In further stratified analyses by prior tumor location, outcomes were poorer in the RCC group than in the LCC or ReC groups, but were similar between the latter two groups. These comparable results between any two of the three groups were in agreement with the outcomes among patients with single CRC [14, 15]. To some extent, it could be speculated that the occurrence of SPCRC does not change the survival pattern of CRC patients. Hildebrand et al. also reported that no significant difference was detected in survival times between patients with and without secondary or multiple primary tumors [23]. They further showed that the presence of SPCRC was not considered as an independent prognostic factor in patients with a prior CRC, as assessed on the basis of direct survival comparisons. Multiple primary tumors are not necessarily associated with a worse outcome, therefore patients should receive curative intent surgery and appropriate follow-up care.

The influence of age on the survival of patients with single CRC has been studied, although the results are not consistent [24–28]. Previous studies have not focused on the impact of age on the outcomes in patients with SPCRC after CRC. The current study identified age as a significant prognostic predictor among SPCRC patients with prior CRC. A younger age was associated with a favorable outcome. In addition to their relatively longer life expectancy, the distinct tumor behavior may also play a role in the better survival of the younger patients, when compared to the older patients. Young patients with multiple primary CRCs usually had a positive family history and were more likely to carry germline mutations, such as the deficient mismatch repair gene [29]. Screening using more accurate biomarkers should help to identify these patients. For this purpose, an intensive biomarker study could be conducted in the future.

Among the patients with SPCRC, the tumor location of prior CRC was not an independent overall prognostic factor. The overall survival difference by tumor location disappeared only when adjusted or stratified by the factor of age. Therefore, the survival difference by tumor location mainly resulted from the special pattern of age distribution in the three location groups. The highest proportion of the patients ≥70 years of age, and the association between older age and unfavorable outcomes, led to the worst outcomes in the RCC survivors. In addition, the CSS percentages were similar among the three location groups, but were different among the five age groups. These results indicated that tumor behavior might vary substantially according to age of prior CRC diagnosis, rather than according to prior tumor location. In the clinical surveillance of CRC survivors, age is therefore a more important factor than tumor location of the prior CRC. Young patients with prior CRC had a higher risk for SPCRC, but a better survival than that of the older patients, because they received standard and curable treatment and had longer follow-up times.

In this retrospective study, a large sample size was reliably identified from the SEER database. All patients received at least a 7 year follow-up until their death, or to the deadline of December, 2012. In our study, the survival of patients with SPCRC after prior CRC was evaluated.

This study had some limitations. First, in some cases, data were lacking concerning the detailed records of patient follow-ups, and concerning the specific advice given to patients about their surveillance methods. Second, data on genetic variations were not included, making it impossible to directly compare our findings with molecular studies. In the future, more detailed genetic variation studies will be required to identify patients at high risk in order to include a better individual clinical surveillance.

## Conclusions

The risk of SPCRC increased among patients with both prior RCC and LCC, but not among those patients with ReC. The differences in survival outcomes in CRC survivors suffering from SPCRC were largely correlated with patient age, but not with prior tumor location. In the clinical surveillance of CRC survivors, age at prior diagnosis is a more important factor than prior CRC location.

## Additional files

Additional file 1: Table S1. Standardized incidence ratio for SPCRC by anatomical sites of index colorectal cancer. (DOCX 27 kb)

Additional file 2: Table S2. Standardized incidence ratio for SPCRC by latency among colorectal cancer survivors. (DOCX 29 kb)

Additional file 3: Table S3 Standardized incidence ratio for SPCRC by age among colorectal cancer survivors. (DOCX 26 kb)

Additional file 4: Table S4. Characteristics of index cancer among patients with single colorectal cancer and patients with SPCRC. (DOCX 25 kb)

Additional file 5:Table S5. Median survival of SPCRC patients according to prior tumor location in stratified subgroups. (DOCX 35 kb)

## Abbreviations

CI: confidence interval
CRC: colorectal cancer
CSS: cancer-specific survival
HR: hazard ratio
LCC: left colon cancer
OR: Odds ratio
OS: overall survival
RCC: right colon cancer
ReC: rectal cancer
SEER: Surveillance, Epidemiology, and End Results Program database
SIR: standardized incidence ratio
SPCRC: subsequent primary colorectal cancer
