# Supplementary material for: The risk and survival outcome of subsequent primary colorectal cancer after the first primary colorectal cancer: cases from 1973 to 2012
Source: BMC Cancer. 2017 Nov 22;17:783. doi: 10.1186/s12885-017-3765-8 (PMC5700626; doi:10.1186/s12885-017-3765-8)
Supplement: Supplementary file 1 — Standardized incidence ratio for SPCRC by anatomical sites of index colorectal cancer. (DOCX 27 kb) [file 12885_2017_3765_MOESM1_ESM.docx]

Table S1. Standardized incidence ratio for SPCRC by anatomical sites of index colorectal cancer

| Tumor site | Observed | % | SIR (95% CI) |
| --- | --- | --- | --- |
| Colorectum | 7290 | 100 | 1.27 (1.24－1.30) |
| Appendix | 22 | 0.3 | 2.31(1.45－3.50) |
| Cecum | 1018 | 14.0 | 1.15(1.08－1.23) |
| Ascending colon | 782 | 10.7 | 1.34 (1.25－1.44) |
| Hepatic colon | 266 | 3.7 | 1.47 (1.30－1.66) |
| Transverse colon | 715 | 9.9 | 1.86 (1.73－2.00) |
| Splenic colon | 279 | 3.8 | 1.85 (1.64－2.08) |
| Descending colon | 510 | 7.0 | 1.65 (1.51－1.80) |
| Sigmoid colon | 1867 | 25.6 | 1.22 (1.17－1.28) |
| Rectosigmoid junction | 732 | 10.0 | 1.19 (1.10－1.27) |
| Rectum | 1024 | 14.0 | 1.00 (0.94－1.06) |
| Large bowel, NOS | 75 | 1.0 | 1.29(1.01－1.61) |

Abbreviations: SPCRC, subsequent primary colorectal cancer; SIR, standardized incidence ratio; CI, confidence interval; NOS, non-specific
