# Supplementary material for: The risk and survival outcome of subsequent primary colorectal cancer after the first primary colorectal cancer: cases from 1973 to 2012
Source: BMC Cancer. 2017 Nov 22;17:783. doi: 10.1186/s12885-017-3765-8 (PMC5700626; doi:10.1186/s12885-017-3765-8)
Supplement: Supplementary file 2 — Standardized incidence ratio for SPCRC by latency among colorectal cancer survivors. (DOCX 29 kb) [file 12885_2017_3765_MOESM2_ESM.docx]

Table S2. Standardized incidence ratio for SPCRC by latency among colorectal cancer survivors

| Latency | All | | RCC | | LCC | | ReC | |
| --- | --- | --- | --- | --- | --- | --- | --- | --- |
|  | Observed | SIR(95% CI) | Observed | SIR(95% CI) | Observed | SIR(95% CI) | Observed | SIR(95% CI) |
| 7-12 | 548 | 1.46(1.34－1.59) | 195 | 1.40(1.21－1.61) | 277 | 1.73(1.53－1.94) | 70 | 1.01(0.79－1.27) |
| 13-24 | 1017 | 1.56(1.47－1.66) | 429 | 1.78(1.62－1.96) | 462 | 1.64(1.49－1.79) | 113 | 0.94(0.78－1.13) |
| 25-36 | 807 | 1.45(1.36－1.56) | 323 | 1.57(1.40－1.75) | 377 | 1.56(1.40－1.70) | 9 | 0.99(0.80－1.20) |
| 37-48 | 715 | 1.48(1.37－1.59) | 284 | 1.58(1.40－1.77) | 330 | 1.56(1.39－1.73) | 87 | 1.01(0.81－1.25) |
| 49-60 | 531 | 1.25(1.14－1.35) | 199 | 1.25(1.08－1.43) | 250 | 1.33(1.17－1.50) | 78 | 1.04(0.82－1.30) |
| 61-72 | 416 | 1.09(0.99－1.20) | 154 | 1.08(0.92－1.27) | 197 | 1.16(1.01－1.34) | 60 | 0.90(0.69－1.16) |
| 73-84 | 371 | 1.08(0.97－1.19) | 139 | 1.10(0.93－1.30) | 17 | 1.16(1.00－1.35) | 50 | 0.83(0.62－1.10) |
| 85-96 | 351 | 1.13(1.02－1.26) | 123 | 1.09(0.91－1.30) | 166 | 1.19(1.10－1.38) | 57 | 1.05(0.80－1.36) |
| 97-108 | 292 | 1.04(0.93－1.17) | 119 | 1.19(0.98－1.42) | 131 | 1.03(0.86－1.22) | 38 | 0.78(0.55－1.07) |
| 109-120 | 291 | 1.16(1.03－1.30) | 109 | 1.22(1.00－1.47) | 130 | 1.13(0.94－1.34) | 46 | 1.05(0.77－1.40) |
| 121-180 | 1041 | 1.14(1.07－1.21) | 389 | 1.26(1.14－1.39) | 472 | 1.09(1.00－1.19) | 162 | 1.00(0.85－1.17) |
| 181+ | 910 | 1.21(1.13－1.29) | 318 | 1.42(1.27－1.58) | 417 | 1.10(0.99－1.21) | 164 | 1.15(0.98－1.34) |

Abbreviations: SPCRC, subsequent primary colorectal cancer; RCC, right colon cancer; LCC, left colon cancer; ReC, rectal cancer; SIR, standardized incidence ratio; CI, confidence interval
