# Supplementary material for: The risk and survival outcome of subsequent primary colorectal cancer after the first primary colorectal cancer: cases from 1973 to 2012
Source: BMC Cancer. 2017 Nov 22;17:783. doi: 10.1186/s12885-017-3765-8 (PMC5700626; doi:10.1186/s12885-017-3765-8)
Supplement: Supplementary file 3 — Standardized incidence ratio for SPCRC by age among colorectal cancer survivors. (DOCX 26 kb) [file 12885_2017_3765_MOESM3_ESM.docx]

Table S3. Standardized incidence ratio for SPCRC by age among colorectal cancer survivors

| Age | All | | RCC | | LCC | | ReC | |
| --- | --- | --- | --- | --- | --- | --- | --- | --- |
|  | Observed | SIR | Observed | SIR | Observed | SIR | Observed | SIR |
| ≤50 | 190 | 7.91 | 72 | 9.84 | 80 | 7.64 | 28 | 4.92 |
| 51-60 | 541 | 2.83 | 194 | 3.62 | 292 | 3.03 | 76 | 1.56 |
| 61-70 | 1423 | 1.47 | 496 | 1.84 | 688 | 1.46 | 219 | 1.02 |
| 71-80 | 2735 | 1.22 | 1025 | 1.39 | 1309 | 1.24 | 370 | 0.87 |
| ≥81 | 2371 | 1.04 | 994 | 1.04 | 1019 | 1.05 | 331 | 0.99 |

Abbreviations: SPCRC, subsequent primary colorectal cancer; RCC, right colon cancer; LCC, left colon cancer; ReC, rectal cancer; SIR, standardized incidence ratio; CI, confidence interval
