# Supplementary material for: The risk and survival outcome of subsequent primary colorectal cancer after the first primary colorectal cancer: cases from 1973 to 2012
Source: BMC Cancer. 2017 Nov 22;17:783. doi: 10.1186/s12885-017-3765-8 (PMC5700626; doi:10.1186/s12885-017-3765-8)
Supplement: Supplementary file 4 — Characteristics of index cancer among patients with single colorectal cancer and patients with SPCRC. (DOCX 25 kb) [file 12885_2017_3765_MOESM4_ESM.docx]

**Table S4**. Characteristics of index cancer among patients with single colorectal cancer and patients with SPCRC

|  | Single Colorectal Cancer (n=195387) | SPCRC  (n=6701) |  |
| --- | --- | --- | --- |
| Age at diagnosis |  |  | <0.0001 |
| ≤ 50 | 18999(9.72) | 474(7.07) |  |
| 51-60 | 32865(16.82) | 1111(16.58) |  |
| 61-70 | 52618(26.93) | 2195(32.76) |  |
| 71-80 | 57617(29.49) | 2150(32.08) |  |
| ≥ 81 | 33288(17.04) | 771(11.51) |  |
| Year of diagnosis |  |  | <0.0001 |
| 1973-1985 | 74904(38.34) | 3434(51.25) |  |
| 1986-1995 | 60019(30.72) | 2206(32.92) |  |
| 1996-2005 | 60464(30.95) | 1061(15.83) |  |
| Race |  |  | <0.0001 |
| white | 166370(85.15) | 5775(86.18) |  |
| Black | 15592(7.98) | 487(7.27) |  |
| Others | 13021(6.87) | 439(6.55) |  |
| Gender |  |  | <0.0001 |
| Female | 97050(49.67) | 3563(53.17) |  |
| Male | 98337(50.33) | 3138(46.83) |  |
| Stage |  |  | <0.0001 |
| Localized | 56971(29.16) | 2970(44.32) |  |
| Regional | 82337(42.14) | 3277(48.9) |  |
| Distant | 46443(23.77) | 261(3.89) |  |
| Unknown | 9636(4.93) | 193(2.88) |  |
| Grade |  |  | <0.0001 |
| Grade I | 19959(10.22) | 868(12.95) |  |
| Grade II | 106181(54.34) | 3634(54.23) |  |
| Grade III | 35216(18.02) | 1010(15.07) |  |
| Unknown | 34031(17.42) | 1189(17.74) |  |
| Tumor location |  |  | <0.0001 |
| RCC | 71001(36.34) | 2572(38.38) |  |
| LCC | 63649(32.60) | 3166(47.25) |  |
| ReC | 60737(31.08) | 963(14.37) |  |

Abbreviations: SPCRC, subsequent primary colorectal cancer; RCC, right colon cancer; LCC, left colon cancer; ReC, rectal cancer.
