# Supplementary material for: The risk and survival outcome of subsequent primary colorectal cancer after the first primary colorectal cancer: cases from 1973 to 2012
Source: BMC Cancer. 2017 Nov 22;17:783. doi: 10.1186/s12885-017-3765-8 (PMC5700626; doi:10.1186/s12885-017-3765-8)
Supplement: Supplementary file 5 — Median survival of SPCRC patients according to prior tumor location in stratified subgroups. (DOCX 35 kb) [file 12885_2017_3765_MOESM5_ESM.docx]

**Table S5.** Median survival of SPCRC patients according to prior tumor location in stratified subgroups

|  | Overall survival (month) | | | |  | Cancer-specific survival (month) | | | |
| --- | --- | --- | --- | --- | --- | --- | --- | --- | --- |
|  | RCC | LCC | ReC | P |  | RCC | LCC | ReC | **P** |
| All | 110(104-115) | 116(110-121) | 125(116-133) | <0.0001 |  | 253(229-276) | 268(247-288) | 243(213-272) | 0.455 |
| Stage |  |  |  |  |  |  |  |  |  |
| Localized | 125 (118-132) | 136 (128-144) | 139 (127-151) | 0.022 |  | 318(285-414) | 312(279-344) | 256(217-294) | 0.115 |
| Regional | 103 (94-112) | 110 (103-117) | 101 (86-116) | 0.010 |  | 215(191-237) | 232(204-259) | 217(159-274) | 0.143 |
| Distant | 43 (32-54) | 51 (42-60) | 52 (33-71) | 0.775 |  | 56(43-68) | 62(41-82) | 60(25-101) | 0.929 |
| Unknown | 131 (93-169) | 84 (62-106) | 115 (90-140) | 0.046 |  | - | 166(122-209) | 211(85-336) | 0.436 |
| Grade |  |  |  |  |  |  |  |  |  |
| I | 124 (108-140) | 127 (112-142) | 145 (116-174) | 0.693 |  | 298(242-353) | 293(233-352) | 327(257-396) | 0.901 |
| II | 100 (93-107) | 108 (102-114) | 115 (104-126) | 0.002 |  | 225(197-252) | 268(239-296) | 234(187-280) | 0.09 |
| III | 110 (97-123) | 115 (98-102) | 128(103-152) | 0.143 |  | 260(222-297) | 232(187-276) | 203(144-262) | 0.294 |
| Unknown | 138 (126-150) | 141 (129-153) | 141 (116-166) | 0.361 |  | 350(274-426) | 285(243-326) | 243(179-307) | 0.545 |
| Race |  |  |  |  |  |  |  |  |  |
| White | 112 (106-118) | 119 (113-125) | 125 (116-134) | <0.0001 |  | 253(229-276) | 276(254-297) | 227(201-252) | 0.191 |
| Black | 83 (66-100) | 88 (74-102) | 99 (58-140) | 0.687 |  | 198(141-255) | 143(99-187) | - | 0.150 |
| Others | 120 (91-149) | 127 (108-146) | 164 (128-200) | 0.700 |  | 304(239-369) | 267(192-341) | 313(239-269) | 0.575 |
| Gender |  |  |  |  |  |  |  |  |  |
| Male | 99 (92-106) | 109 (104-114) | 117 (106-128) | 0.011 |  | 247(213-281) | 244(220-267) | 253(221-284) | 0.452 |
| Female | 124 (116-132) | 134 (125-143) | 135 (118-152) | 0.001 |  | 264(232-296) | 285(255-314) | 219(188-249) | 0.454 |
| Year of diagnosis |  |  |  |  |  |  |  |  |  |
| 1973-1985 | 140 (133-147) | 151 (144-158) | 164 (150-178) | 0.002 |  | 297(263-330) | 308(281-334) | 293(264-322) | 0.372 |
| 1986-1995 | 107 (98-116) | 103 (97-109) | 113 (103-123) | 0.768 |  | 233(198-267) | 231(196-265) | 198(171-224) | 0.514 |
| 1996-2005 | 59 (55-63) | 66 (61-71) | 65 (58-72) | 0.327 |  | 135(89-180) | 113(90-135) | 115(80-150) | 0.870 |
| Age at diagnosis |  |  |  |  |  |  |  |  |  |
| ≤ 50 | 248 (225-271) | 206 (159-252) | 231 (170-292) | 0.779 |  | - | 320(254-385) | - | 0.053 |
| 51-60 | 153 (131-175) | 172 (155-189) | 178 (153-203) | 0.295 |  | 304(247-360) | 334(295-372) | 256(216-295) | 0.344 |
| 61-70 | 137 (127-147) | 140 (132-148) | 144 (127-161) | 0.427 |  | 302(266-337) | 284(253-314) | 291(256-325) | 0.965 |
| 71-80 | 96 (88-104) | 94 (87-101) | 97 (86-108) | 0.458 |  | 195(167-222) | 192(161-222) | 152(121-182) | 0.452 |
| ≥ 81 | 59 (54-64) | 63 (55-71) | 58 (51-65) | 0.225 |  | 126(92-159) | 111(83-138) | 96(63-129) | 0.470 |

Abbreviations: SPCRC, subsequent primary colorectal cancer; RCC, right colon cancer; LCC, left colon cancer; ReC, rectal cancer.
